# The features of polyglutamine regions depend on their evolutionary stability

**DOI:** 10.1186/s12862-020-01626-3

**Published:** 2020-05-24

**Authors:** Pablo Mier, Miguel A. Andrade-Navarro

**Affiliations:** Institute of Organismic and Molecular Evolution, Faculty of Biology, Johannes Gutenberg University Mainz, Hanns-Dieter-Hüsch-Weg 15, 55128 Mainz, Germany

**Keywords:** Homorepeat, polyQ, Glutamine, Codon usage, Evolutionary stability

## Abstract

**Background:**

Polyglutamine regions (polyQ) are one of the most studied and prevalent homorepeats in eukaryotes. They have a particular length-dependent codon usage, which relates to a characteristic CAG-slippage mechanism. Pathologically expanded tracts of polyQ are known to form aggregates and are involved in the development of several human neurodegenerative diseases. The non-pathogenic function of polyQ is to mediate protein-protein interactions via a coiled-coil pairing with an interactor. They are usually located in a helical context.

**Results:**

Here we study the stability of polyQ regions in evolution, using a set of 60 proteomes from four distinct taxonomic groups (Insecta, Teleostei, Sauria and Mammalia). The polyQ regions can be distinctly grouped in three categories based on their evolutionary stability: stable, unstable by length variation (inserted), and unstable by mutations (mutated). PolyQ regions in these categories can be significantly distinguished by their glutamine codon usage, and we show that the CAG-slippage mechanism is predominant in inserted polyQ of Sauria and Mammalia. The polyQ amino acid context is also influenced by the polyQ stability, with a higher proportion of proline residues around inserted polyQ. By studying the secondary structure of the sequences surrounding polyQ regions, we found that regarding the structural conformation around a polyQ, its stability category is more relevant than its taxonomic information. The protein-protein interaction capacity of a polyQ is also affected by its stability, as stable polyQ have more interactors than unstable polyQ.

**Conclusions:**

Our results show that apart from the sequence of a polyQ, information about its orthologous sequences is needed to assess its function. Codon usage, amino acid context, structural conformation and the protein-protein interaction capacity of polyQ from all studied taxa critically depend on the region stability. There are however some taxa-specific polyQ features that override this importance. We conclude that a taxa-driven evolutionary analysis is of the highest importance for the comprehensive study of any feature of polyglutamine regions.

## Background

Polyglutamine regions (polyQ) are homorepeats defined by a higher local proportion of glutamines than expected in protein sequences. Due to this relaxed definition, multiple combinations of glutamines and non-glutamine residues (impurities) may form a polyQ. A minimum of four glutamines in a six-amino acid region are nonetheless required [1].

PolyQ are undoubtedly the most studied homorepeat for various reasons. First of all, it is one of the most prevalent homorepeats in eukaryotic proteomes [2–5]. Second, from the point of view of the nucleotide composition of the corresponding coding region, it is a relatively simple homorepeat to study, as glutamine is coded by only two codons, CAG and CAA. Third, they are associated to ten neurodegenerative diseases [6], elicited by CAG expansions. And last, it is one of the few homorepeats for which an expansion mechanism has been proposed, a replication-mediated slippage [7].

As a result, much is known about these homorepeats. Almost non-existent in archaea and bacteria, they are widely present in eukaryotic species [5, 8]. Codon usage is species- and polyQ-length dependent, but they are usually highly enriched in CAG over CAA; as an example, in mammals they are coded by a 3:1 proportion of CAG:CAA [9]. They can be found in multiple lengths and with variable quantities of impurities within [10]. The association between polyQ length and impurity distribution is highly species-dependent, but generally shorter pure polyQ prevail. Regarding impurities, leucine and proline residues are common in impure polyQ [10]. Both these residues also play a role in the amino acid context of some polyQ regions. The former is enriched in position − 1 of polyQ in vertebrates [10]; it has been proposed that it promotes an alpha-helical structure towards the polyQ, making it less aggregation prone [11, 12]. The latter follows long human polyQ in the form of a proline-rich or polyP region [13], also to limit the aggregation propensity of the long polyQ [14, 15]. These sequence mechanisms surrounding polyQ regions are in place to attenuate a polyQ length-dependent aggregation process, which in humans ultimately leads to disease [16]. Protein aggregation in this case is a byproduct of an abnormally long polyQ that has to do with its wild-type biological role in stabilizing protein-protein interactions [13]; new abnormal interactions may be gained by the extended polyQ resulting in aggregates. Regarding the structural conformation of polyQ regions, it has been described that they are preceded by a helical conformation and followed by random coil [1].

It has been theorized that there are two possible ways for a polyQ region to emerge in a sequence: by replication slippage and by point mutations [7]. Albà and colleagues described the different codon usage of polyQ regions from seven model organisms based on these mechanisms. Whether any other polyQ feature is influenced by its emergence mechanism is yet to be described. To the best of our knowledge, no other study since then has tried to take these mechanisms into account when studying polyQ regions or any other homorepeat. Our aim is to challenge the established knowledge about polyQ features and to classify polyQ based on the way they emerged (or are emerging) in a sequence. While they use the nucleotide sequence to classify the regions, our take is to classify them based on the amino acid sequence, and to establish whether a polyQ is stable or unstable in evolution and how the degree of stability relates to their amino acid sequence and function.

Here we use the proteomes of 60 species from four different taxa (Class Insecta, Infraclass Teleostei, Clade Sauria and Class Mammalia) to generate sets of orthologs of polyQ-containing proteins. Then we classify the orthologous polyQ regions in three categories based on their stability, that is, general conservation of the glutamine residues. Nearly all of the polyQ features described above are studied based on these three polyQ stability categories. We show how all known features are category-dependent, and thus evolutionary information is needed to establish general rules as to the behavior of polyQ regions.

## Results

### PolyQ regions can be categorized based on their evolutionary stability

A protein sequence is thought to be something static, a fixed ordered set of amino acids reflecting the biological entity that is a protein. But in reality, the same sequence is constantly under selective pressure that may be favoring sequence alterations. Here we aim to study whether polyQ regions can be categorized based on their evolutionary stability. We define this stability comparing polyQ regions across orthologs from taxonomically close species.

**Fig. 1:**
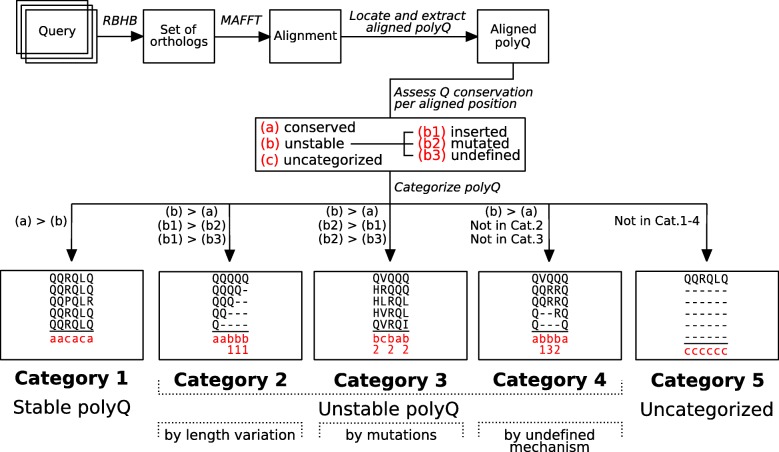
Pipeline for polyQ categorization. Simplification of steps followed to categorize polyQ regions based on their stability when compared to other orthologous regions. RBHB: Reciprocal Best-Hit BLAST

We prepared aligned sets of orthologs in which at least one protein has a polyQ region (see Methods for details), for four distinct taxonomic groups: Class Insecta, Infraclass Teleostei, Clade Sauria and Class Mammalia. The aligned polyQ regions were then studied in detail to assess the glutamine conservation per aligned position (Fig. 1). Each position has three possible values, depending on the proportion of sequences having a glutamine:

- conserved, if > = 80% of the sequences have a glutamine;
- unstable, if < 80% but > = 20% of the sequences have a glutamine; and
- uncategorized, if < 20% of the sequences have a glutamine.

For (a) and (c) it is clear that the glutamine in that position is clearly conserved or absent in the alignment, respectively. In the case of (b), we further defined three sub-values:

1. inserted, if there are more gaps than non-glutamine amino acids;
2. mutated, if there are more non-glutamine amino acids than gaps;
3. undefined, if the number of gaps and non-glutamine amino acids is the same.

The (b) value reflects an aligned position not dominated by glutamines, being the sub-value an indicator of the possible underlying mechanism driving the position. More gaps would mean a length variation in the sequence, whereas any residue different to glutamine would imply a point mutation either to or from the glutamine.

The stability of the polyQ is categorized by taking into consideration all the values of glutamine conservation along the complete span of the polyQ region. Five categories are defined:

- Category 1, stable polyQ. More (a) conserved than (b) unstable positions.
- Category 2, unstable polyQ by length variation. More (b) unstable than (a) conserved positions, and more (b1) inserted than (b2) mutated and (b3) undefined.
- Category 3, unstable polyQ by mutations. More (b) unstable than (a) conserved positions, and more (b2) mutated than (b1) inserted and (b3) undefined.
- Category 4, unstable polyQ by undefined mechanism. More (b) unstable than (a) conserved positions, and not in category 2 or 3.
- Category 5, uncategorized. Not in any previous category.

Although five categories are defined, only in 1, 2 and 3 it seems clear how the stability or instability of the polyQ region is achieved in the orthologs. In category 4 it is theoretically not possible to determine whether the instability comes from an *indel* or a point mutation, and category 5 is by definition place for the uncategorized regions. For the purpose of the present work we discard hereafter the polyQ regions in categories 4 and 5, and refer to the selected categories as stable (1), inserted (2) and mutated (3).

**Fig. 2:**
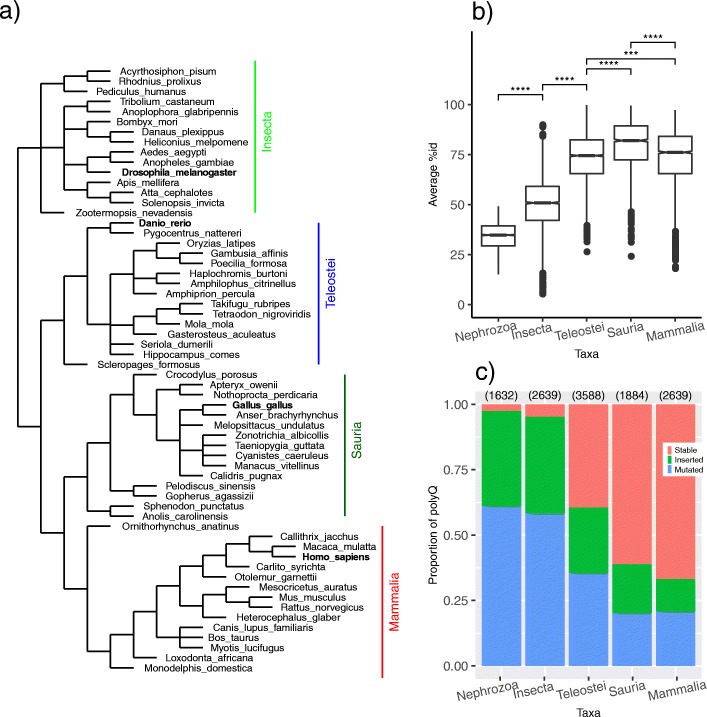
Species selection and categorization of polyQ regions. **a** Cladogram of the 60 species used in the present work, 15 per taxon (Insecta, Teleostei, Sauria and Mammalia); in bold, the species taken as representative per taxa to compute taxa Nephrozoa. **b** Average %identity of proteins in sets of orthologs from different taxa. **c** Proportion of polyQ per category per taxa; categories are defined as stable, inserted or mutated polyQ; on top, number of polyQ cases per taxa

Stating that a polyQ is stable means that it is conserved in at least 80% of the orthologs. It is implied that the categorization of the polyQ is intrinsically affected by the selection of the species to use in the study (Fig. 2a), as more similar orthologs would mean more stable polyQ. However, this relation is not straightforward, and the sequence similarity in the orthologs (Fig. 2b) is not directly correlated with having more stable polyQ regions (Fig. 2c). It is interesting to note that Mammalia has a higher proportion of stable polyQ even compared with classes Teleostei and Sauria, for which we obtained orthologs similarly conserved. The difference to class Insects could be due to the higher divergence of the set of insect orthologs resulting in more polyQ identified as mutated or inserted. Nonetheless, with our choice of species the number of polyQ regions per taxa is high enough for our purposes of obtaining statistically significant results (2639, 3588, 1884, 2639 in Insecta, Teleostei, Sauria and Mammalia, respectively). For the record, the number of discarded polyQ (categories 4 and 5) was of 1496, 740, 359, and 388 in Insecta, Teleostei, Sauria and Mammalia, respectively.

We included results for the Nephrozoa taxa, taking one representative species from each of the taxa considered above (see Methods for details), to show the effect of lower sequence similarity when using distant orthologs (Fig. 2b) and, as a result, the minimal number in stable polyQ compared to unstable ones (Fig. 2c).

### Glutamine codon usage depends on polyQ stability category

Glutamine codon usage is biased towards CAG over CAA codons, especially in polyQ regions of Vertebrata species (3:1 proportion) [9]. Here we intend to determine whether there is a difference in this behavior based on the stability category of the polyQ region. To this end, we studied the codon usage in all glutamines within the orthologous polyQ regions. This means that not all considered glutamines are necessarily part of a polyQ, but they are in at least one of the orthologs. Even if they are not forming a polyQ themselves, placing them in an evolutionary context by studying their orthologs could show whether they have sequence or structural features in consonance with forming a polyQ.

**Fig. 3:**
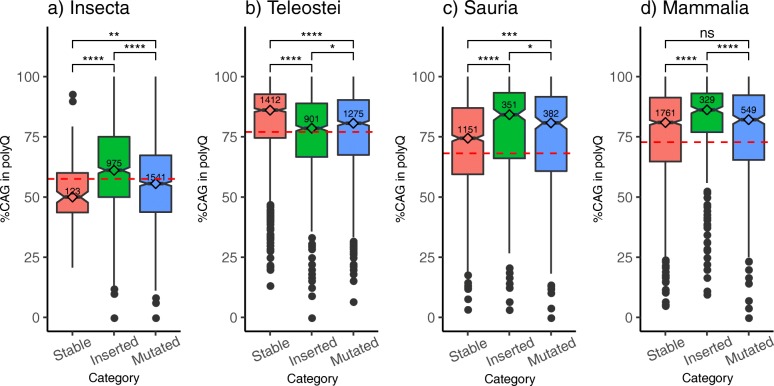
Glutamine codon usage in polyQ regions. %CAG codons in polyQ regions of orthologous sets of proteins from **a**) Insecta, **b**) Teleostei, **c**) Sauria and d) Mammalia. Categories are defined as: stable polyQ; inserted polyQ; mutated polyQ. Non-parametric Mann-Whitney U statistical tests were performed to compare the distributions (**** *P*-value <= 1e-4; *** P-value <= 1e-3; ** P-value <= 0.01; * P-value <= 0.05; ns = not significant). Number of sets of polyQ regions considered per category are shown within each boxplot

PolyQ regions in the chordate taxa (Teleostei, Sauria and Mammalia) are significantly enriched in CAG codons, close or slightly above the background codon ratio CAG to CAA of 3:1, as expected (Fig. 3b-d). Differently, glutamine codon usage in polyQ from Insecta does not differ much from their background, which is close to 1:1 (Fig. 3a); an unexpected decrease in the %CAG is even apparent in stable polyQ. According to the values of significance comparing the distributions, the most significant differences are higher %CAG in inserted polyQ in Insecta and Mammalia, and different %CAG in stable polyQ in Teleostei (higher) and in Sauria (lower). Separation above the background is higher in Sauria and Mammalia than in Insecta and Teleostei, with highest %CAG in inserted polyQ. This is in line with previous results showing that trinucleotide repeats are subject to a CAG-slippage mechanism [17].

The categories of polyQ stability can be significantly distinguished by their %CAG, but the differences depend on the taxa. Results suggest that the CAG-slippage mechanism for which polyQ regions vary in length are predominant in inserted polyQ in Amniota species (comprising taxa Sauria and Mammalia).

### The polyQ amino acid context is influenced by polyQ stability

Next, we study the sequences surrounding the polyQ regions. In this respect, previous work have described how polyQ regions are usually followed by a proline-rich or polyP region [13]. In addition, we recently described an unusual peak of leucine residues in position − 1 of polyQ regions from several taxonomically diverse species [10]. Here, we took one sequence at random from each set of orthologous regions, but forcing the selected sequence to have a polyQ. We then calculated the proportion of both leucine and proline residues in the previous (from position − 10 to − 1) and following (from position + 1 to + 10) regions.

**Fig. 4:**
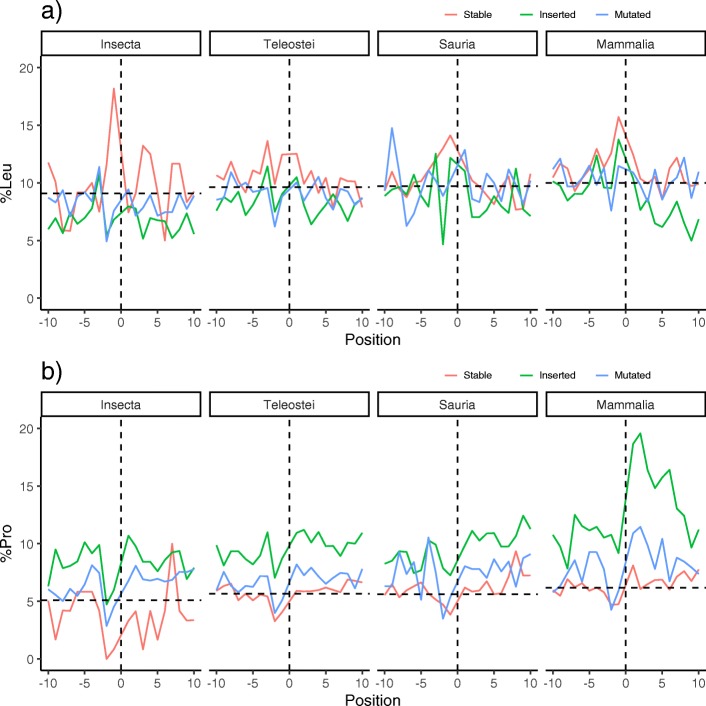
Amino acid context of polyQ regions. **a**) Leucine and **b**) proline abundance around polyQ regions (positions − 10 to + 10) depending on their category and taxa. Vertical lines refer to the position of the polyQ, and horizontal lines to the background composition of the amino acid per taxa

Although the results do not seem to be steady for leucine residues in any of the taxa, the highest proportion in position − 1 is always achieved in stable polyQ (Fig. 4a). This is especially clear for mammalian sequences. It must be noted that it is difficult to draw conclusions from the noisy results from Insecta sequences due to the low amount of sequences considered (123 stable polyQ, versus, for example, 1761 stable polyQ in Mammalia). We also note that the threshold used in this work to look for polyQ regions, 4/6, results in a lower signal for leucines in position − 1 than other thresholds (4/4, 6/6, 6/8, 8/10) when examining human proteins [10]. While the 4/6 threshold maximizes the number of polyQ found, features specific to longer polyQ may be obscured by the larger number of short polyQ.

Almost the complete N-terminal and C-terminal surrounding sequences of inserted polyQ regions in the four taxa are enriched in prolines, specially C-terminally to polyQ in mammalian sequences (Fig. 4b). The signal is also present to some extent in mutated polyQ. The C-terminal capping of polyQ regions has been proposed as a protective mechanism against the aggregation they induce [14]. As aggregation propensity is polyQ-length dependent [16], it is not surprising that inserted polyQ (which is defined by having varying length) is the category clearly showing this position-specific enrichment. We observed a remarkable decay in proline frequency in position − 2, shared by all categories in all taxa. Prolines in that position would difficult the function of polyQ in expanding a preceding alpha-helix upon protein interaction, since prolines act as helix breakers.

### Stable polyQ have higher tendency to be preceded by helical structure

We previously reported that polyQ regions have a tendency to be preceded by a sequence in helical conformation and followed by a region in random coil conformation [1]. Following the strategy of previous sections, and assuming that the secondary structure of all orthologous sequences will be similar, we predicted the secondary structure of one protein per set of orthologs using the tool JPred [18]. More specifically, we used as input sequence the polyQ region plus its sequence context. JPred uses a neural network to classify each residue to be structured as alpha helix (helical), beta sheet (extended) or as other secondary structure.

**Fig. 5:**
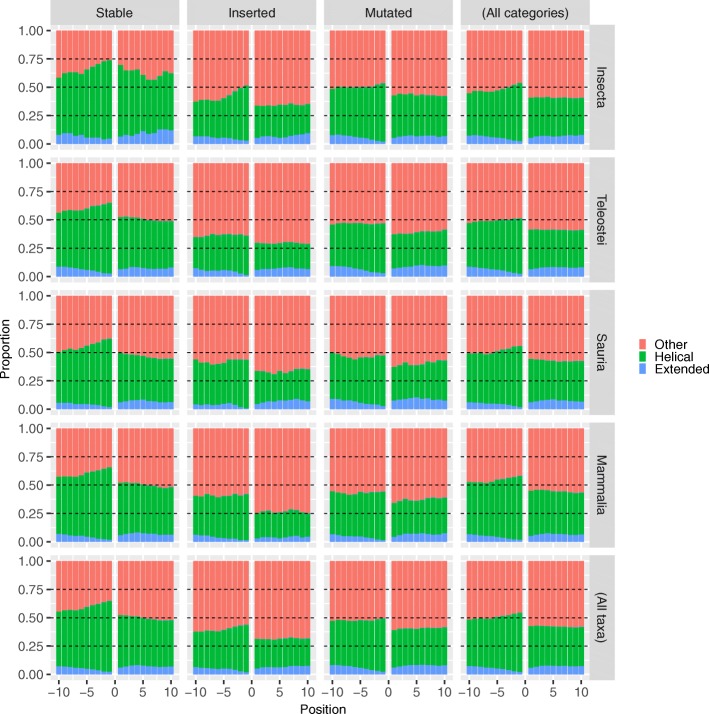
Secondary structure prediction around polyQ regions. Proportion of predicted alpha helical (green; ‘Helical’), beta sheet (blue; ‘Extended’) and other (red; ‘Other’) secondary structure conformation in the N- (from position − 10 to − 1) and C- (from position + 1 to + 10) terminal regions of polyQ. Results were calculated for proteins in the three polyQ categories and taxa Insecta, Teleostei, Sauria and Mammalia

In all polyQ categories and taxa there is higher helical conformation content N-terminally to the polyQ (with a steep increase from the N-terminal side for stable polyQ; Fig. 5). The aggregation of structural predictions by taxa (last column) and by category (bottom row) highlights that the category in which a polyQ is classified (stable, inserted or mutated) is more relevant than the taxa with respect to its surrounding secondary structure.

Using the results given by JPred we also studied the solvent accessibility of the residues in the input sequences. However, we found no differences between taxa or categories (data not shown). Similarly, we predicted the coiled coil propensity of the sequences using DeepCoil and also found no significant differences (data not shown).

### The protein-protein interaction capacity of a polyQ is affected by its stability

PolyQ regions are associated to protein-protein interactions (PPIs) [13]. Propensity of a polyQ region to interact may be related to its stability category, as the structural differences described in the previous section may play a role in its interaction capacity. We considered all proteins from the sets of orthologs, independently of whether they or their orthologs have the polyQ region, and calculated the number of high confidence interactors per protein described in the STRING database. We discarded the proteins for which STRING had no entry as we could not distinguish whether they have indeed no interactors or the protein is missing in the database.

**Fig. 6:**
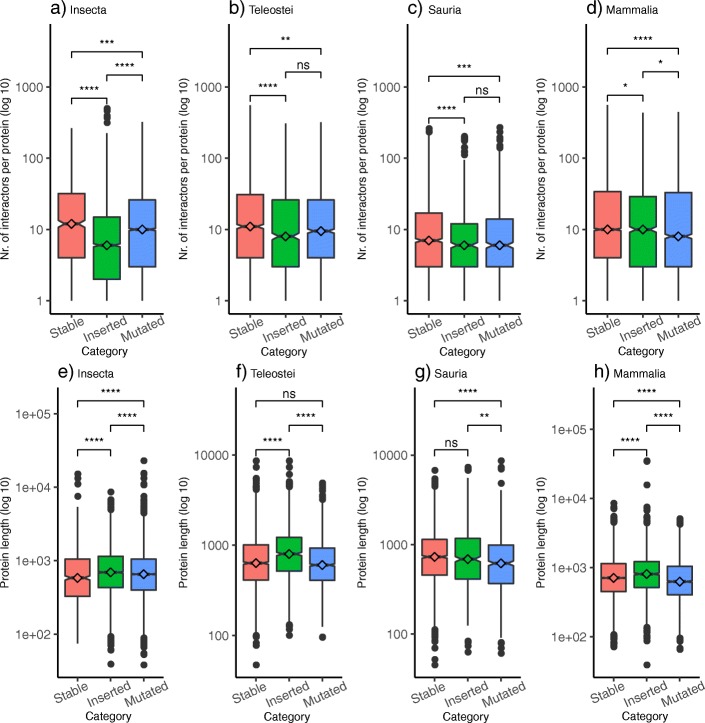
Number of high-confidence interactors per protein. In proteins with at least one interactor. Results are split by polyQ category. Proteins from **a**) Insecta, **b**) Teleostei, **c**) Sauria and **d**) Mammalia. The length distribution of these proteins are represented for **e**) Insecta, **f**) Teleostei, **g**) Sauria and **h**) Mammalia. Non-parametric Mann-Whitney U statistical tests were performed to compare the distributions (**** P-value <= 1e-4; *** P-value <= 1e-3; ** P-value <= 0.01; * P-value <= 0.05; ns = not significant)

Results show that stable polyQ are present in proteins that have significantly and consistently in all taxa more interactors than proteins with unstable polyQ (Fig. 6a-d). As longer proteins tend to have more interactions [13], we calculated the protein length distribution of the proteins to rule out the possibility that our results would be due to stable polyQ being predominant in longer proteins: this is not the case (Fig. 6e-h). Stable polyQ do then have greater number of interactors than unstable polyQ and this is not influenced by the protein length. However, proteins with many interactors are more conserved and this could explain the results.

### Functional differences of polyQ categories

We have studied so far how the stability of a polyQ region affects its features on the nucleotide (codon usage), amino acid (sequence context), structural (secondary structure) and interaction (PPI) level. Lastly, we aim to check whether the polyQ functionality is dependent on its stability. For this purpose, we performed GO enrichment analyses of all proteins from the sets of orthologs for a representative species per taxa. Analyses were done individually per category and species.

**Fig. 7:**
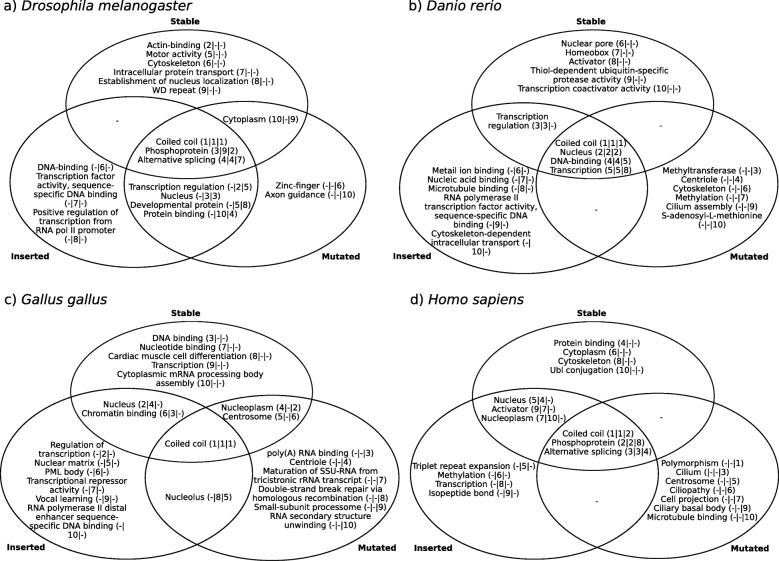
GO enrichment of proteins in the three polyQ categories. Top-10 enriched GO terms per category in proteins from **a**) *Drosophila melanogaster*, **b**) *Danio rerio*, **c**) *Gallus gallus* and d) *Homo sapiens*, as representatives of Insecta, Teleostei, Sauria and Mammalia, respectively. Following each GO term we show its position in the top-10 per category in the format ‘(position in category stable | position in category inserted | position in category mutated)’; a ‘-’ if the term is not present in the top-10 of the category

To ease the interpretation of results, we extracted the top-10 enriched GO terms per dataset and analyzed the commonalities between categories within each species (Fig. 7). Not surprisingly, the term ‘coiled coil’ is the top enriched term in almost all studied conditions since polyQ are known to mediate coiled-coil interactions [13]. More interesting is the enrichment of the term ‘triplet repeat expansion’ exclusively for inserted polyQ of human proteins, pointing towards the correct classification of proteins depending on the polyQ categories. Regarding subcellular location, while stable polyQ are enriched in cytoplasm and nucleus, inserted polyQ are enriched only in nucleus, and mutated polyQ in cilium. Enriched terms related to signaling specifically observed for the human stable polyQ dataset and not for those of the other species (e.g. ‘protein phosphorylation’, Suppl. File 1) could account for the higher proportion of stable polyQ observed in mammals (Fig. 2c), and are consistent with the enrichment in cytoplasmic location.

Analyses were performed independently per species, with sets of proteins specific per taxa, and as such results should not necessarily be similar between species. However, it is interesting to see how some of them are comparable. As an example, GO terms related to ‘transcription’ are mostly limited to inserted polyQ. On the other hand, ‘cilium’ and ‘axon’ related terms are enriched only in mutated polyQ; both cellular structures are known to share proteins [19].

**Fig. 8:**
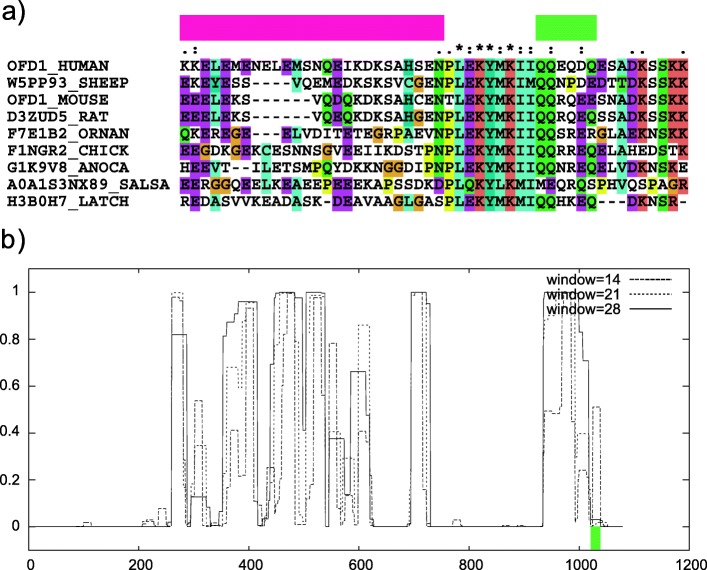
Human OFD1 has a mutated polyQ region at the C-terminal of a predicted coiled coil. **a** Part of a multiple sequence alignment of human OFD1 with orthologs in vertebrate species. The end of a predicted coiled coil region (as annotated in the UniProt entry) and the mutated polyQ region are indicated with the magenta and green boxes, respectively. **b** Coiled coil prediction from COILS for human OFD1. The position of the polyQ is indicated with a green box

We illustrate the presence of mutated polyQ in human proteins annotated with the term ‘cilium’ with the OFD1 protein (UniProtKB ID: O75665) encoded by the *Ofd1* gene (Oral-facial-digital syndrome 1). This protein is required for the formation of primary cilia [20]. It is located in the distal centriole, at the cilium-centriole interface, and controls centriole length [21]. To examine the structural context of the mutated polyQ regions in this protein and the other proteins annotated with the GO term ‘cilium’ (20 proteins in total), we searched for solved structures of the proteins or their homologs (using the web tool Aquaria [22]). Unfortunately, there was no information for or near the polyQ regions. For OFD1 we created a multiple sequence alignment including homologous and orthologous proteins of the human OFD1 chosen from selected organisms, assisted by ProteinPathTracker [23] (Fig. 8a). The polyQ region (‘QQEQDQ’, amino acid positions 965–971) is aligned in a block with gaps but with variable Q content. The position is very close to the C-terminal of a coiled region (Fig. 8b), which is consistent with the function expected for polyQ.

## Discussion

We have shown that there is a relation between polyQ stability and their sequence and structural context. The stability state of a polyQ region is here defined as the divergence of the sequence compared to close orthologs. A stable ‘fixed in evolution’ polyQ may mean that there are sequence or structure constraints that prevent it to vary in length or mutating. While this definition of stability depends on the orthologs used for comparison, in our study we appreciated sequence and structure context signals in stable polyQ that do not depend on the conservation of the orthologous set used.

Our results suggest that while there are differences between the taxa considered, stable polyQ would be generally the one more associated to a strong helical signal, both in sequence context (largest frequency of leucine at − 1 position and depletion of proline at − 2 position; Fig. 4) and structure context (C-terminal steep gradient of helical content; Fig. 5).

We have tried to associate global protein properties (number of protein interactors, and function) to the presence of a stable, inserted or mutated polyQ, which is a local feature. We are aware that this comparison is intrinsically problematic because for example, a protein with many interactions could be more constrained in evolution, resulting in having more stable polyQ (Fig. 6). Similarly, the heterogeneity observed in the functional terms enriched in proteins with inserted or mutated polyQ could be also influenced by different evolutionary restrictions affecting proteins with particular functions (Fig. 7).

It is reasonable to expect that longer proteins will be able to accommodate insertions and deletions more easily. This is consistent with our finding that inserted polyQ are more frequent in longer proteins (Fig. 6e-h). Then, given shorter proteins, those interacting with multiple proteins competing for the same interface (for example, nuclear protein transporters that recognize multiple cargo proteins) will not be able to modify their interacting interface because it would require modifications in all proteins using it. Differently, shorter proteins using specific interfaces may be able to accommodate a polyQ even if structurally constrained to fit some length requirement because mutations can be compensated in the single target protein specifically using the interface. This would explain why stable polyQ is more frequent in proteins with more interactions than those with mutated polyQ.

## Conclusions

We have progressed in the last few years in the knowledge of several aspects of polyQ regions [1, 9, 10, 13]. Here we have shown that, however, there is still much to study about them. To the best of our knowledge this is the first attempt in categorizing any type of homorepeat region based on sequence data but critically depending on an evolutionary perspective. Identical homorepeat sequences may have different sequence features, because, at least for polyQ regions, it is not just the simple concatenation of glutamine residues the important factor but the stability of the region in which it is placed. The question remains as to whether this is a distinct behavior of polyQ regions or a general feature of all homorepeats.

## Methods

### Data retrieval

Protein and CDS sequences were downloaded from the FTP sites of Ensembl release-97 and Ensembl Metazoa release-44 [24] for a set of 60 species (Suppl. File 2).

Taxonomic information for these species was downloaded from the NCBI Taxonomy resource Common Taxonomy Tree [25]. A taxonomic tree using this information was built using the R library phyloseq version 1.29.0 [26].

Protein-protein interaction information was downloaded from the database STRING version 11.0 [27]. Information was parsed so that we only take into account high-confidence interactions (score > 0.7).

### Generation of sets of orthologous polyQ regions

We took all protein sequences per proteome and performed Reciprocal Best Hits BLAST (RBHB) searches [28] with an in-house script (Suppl. File 3) and default parameters against the rest of the proteomes in the same taxa, one at a time, to find an orthologous sequence in the maximum number of proteomes. At least five species must have an ortholog to keep the orthologous set. An exception is made to generate the orthologous sets of the taxa Nephrozoa, comprising representative species *Homo sapiens*, *Gallus gallus*, *Danio rerio* and *Drosophila melanogaster*, in which an ortholog for each of the four species is mandatory. To avoid redundancy, a protein was allowed to be in only one orthologous set. Orthologous sets were then aligned with MAFFT v7.310 with default parameters [29].

The alignments of orthologous sequences were scanned to look for polyQ regions, using a 4/6 threshold (a minimum of four glutamines in a window of six amino acids). This threshold was selected to detect the maximum number of polyQ regions following previous work [1, 10]. Orthologous regions in which at least one sequence had a polyQ were extracted from the alignments and annotated as orthologous polyQ regions. They were studied independently, making it possible to have more than one of these regions per alignment.

Pairwise-distance matrices were calculated by ClustalOmega v.1.2.4 [30] with the alignments of the orthologous sets generated by MAFFT. An average percentage of identity per alignment was calculated for each set of orthologs.

### Secondary structure prediction

Assuming that the secondary structure of a protein is the same in all orthologs, we take one sequence at random per aligned polyQ and extract the region comprising the twenty previous amino acids (− 20 to − 1), the polyQ, and the twenty following amino acids (+ 1 to + 20). We obtained 13,704 sequences following this procedure (4121 in Insecta, 4321 in Teleostei, 2238 in Sauria and 3024 in Mammalia). The secondary structure and the solvent accessibility of these sequences were predicted using a schedule mass submission in JPred RESTful API v.1.5 [31]. Results are plotted from position − 10 to position + 10 to avoid border effects in the secondary structure sequence predictions.

For the same set of sequences we predicted their coiled coil propensity using DeepCoil [32], with a cutoff of > = 0.5 per residue.

### GO enrichment analysis

One model organism per taxa was selected to perform the GO enrichment analyses: *Drosophila melanogaster*, *Danio rerio*, *Gallus gallus* and *Homo sapiens*. All proteins from the set of orthologs from these four species were retrieved, and their Ensembl protein ID were mapped to either UniProt accession numbers using the ID mapping resource provided by UniProt [33] (*D. rerio*, *G. gallus* and *H. sapiens*) or to FlyBase gene ID using the Convert ID tool provided by the FlyBase database version FB2019_04 [34] (*D. melanogaster*). The mapped IDs were distributed in independent files depending on the category of the polyQ they contain (or at least one of their orthologs). These ID lists were used to perform the GO enrichment analyses in DAVID v6.8 [33] per organism and per category. Number of IDs successfully mapped to DAVID IDs per polyQ category (1–3) were: 115, 785, and 1267 for *D. melanogaster*; 978, 562, and 890 for *D. rerio*; 562, 137, and 163 for *G. gallus*; and 1369, 264, and 488 for *H. sapiens*. Selected annotations were UP_KEYWORDS, GOTERM_BP_DIRECT, GOTERM_CC_DIRECT and GOTERM_MF_DIRECT. Duplicated enriched GO terms (e.g. “GP:0005737 ~ cytoplasm” and “Cytoplasm”) were manually simplified to keep only one result. Only the top-10 enriched GO terms per species and categories were taken into account, for simplification purposes; all of them with *p*-values < 0.05.

## Supplementary information

**Additional file 1 : Supplementary File 1**. GO enrichment analysis. Results of the GO enrichment analyses per category and taxa.

**Additional file 2 : Supplementary File 2.** Dataset information for the used species. List of the 60 species used in the study, including the dataset name and number of proteins and CDS downloaded from Ensembl.

**Additional file 3 : Supplementary File 3.** Scripts developed for the analyses. Set of Perl scripts used for the generation, analysis and parsing of results divided by the section of the manuscript in which they are used.

## Data Availability

The datasets used and/or analysed during the current study are available from the corresponding author on reasonable request.

## Abbreviations

PPIs: protein-protein interactions
RBHB: Reciprocal Best Hits BLAST
