# Supplementary material for: The features of polyglutamine regions depend on their evolutionary stability
Source: BMC Evol Biol. 2020 May 24;20:59. doi: 10.1186/s12862-020-01626-3 (PMC7247214; doi:10.1186/s12862-020-01626-3)
Supplement: Supplementary file 2 — Additional file 2 : Supplementary File 2. Dataset information for the used species. List of the 60 species used in the study, including the dataset name and number of proteins and CDS downloaded from Ensembl. [file 12862_2020_1626_MOESM2_ESM.pdf]

Taxa	Species	Dataset	Nr.Proteins	Nr.CDS
Insecta	Acyrthosiphon pisum	Acyr2.0	36195	36195
Insecta	Aedes_aegypti	AaegL3	17158	17158
Insecta	Anopheles gambiae	AgamP4	14916	14916
Insecta	Anoplophora glabripennis	Agla1.0	22343	22343
Insecta	Apis mellifera	Amel4.5	15314	15314
Insecta	Atta cephalotes	Attacep1.0	10657	10657
Insecta	Bombyx mori	ASM15162v1	14623	14623
Insecta	Danaus plexippus	Dpv3	15128	15128
Insecta	Drosophila melanogaster	BDGP6.22	30504	30504
Insecta	Heliconius melpomene	Hmel1	12829	12829
Insecta	Pediculus humanus	PhumU2	10788	10788
Insecta	Rhodnius prolixus	RproC3	15078	15078
Insecta	Solenopsis invicta	Si_gnG	21118	21118
Insecta	Tribolium castaneum	Tcas5.2	18534	18534
Insecta	Zootermopsis nevadensis	ZooNev1.0	14610	14610
Teleostei	Amphilophus citrinellus	Midas_v5	31765	31765
Teleostei	Amphiprion percula	Nemo_v1	34985	34985
Teleostei	Danio rerio	GRCz11	52089	52089
Teleostei	Gambusia affinis	ASM309773v1	32756	32756
Teleostei	Gasterosteus aculeatus	BROADS1	27576	27576
Teleostei	Haplochromis burtoni	AstBur1.0	35619	35619
Teleostei	Hippocampus comes	H_comes_QL1_v1	28435	28435
Teleostei	Mola mola	ASM169857v1	29015	29015
Teleostei	Oryzias latipes	hni.ASM223471v1	35451	35451
Teleostei	Poecilia formosa	PoeFor_5.1.2	30898	30898
Teleostei	Pygocentrus nattereri	Pygocentrus_nattereri-1.0.2	37995	37995
Teleostei	Scleropages formosus	ASM162426v1	38056	38056
Teleostei	Seriola dumerili	Sdu_1.0	33187	33187
Teleostei	Takifugu rubripes	FUGU5	28679	28679
Teleostei	Tetraodon nigroviridis	TETRAODON8	23118	23118
Sauria	Anolis carolinensis	AnoCar2.0	19176	19176
Sauria	Anser brachyrhynchus	ASM259213v1	27104	27104
Sauria	Apteryx owenii	aptOwe1	28116	28116
Sauria	Calidris pugnax	ASM143184v1	29936	29936
Sauria	Crocodylus porosus	CroPor_comp1	27731	27731
Sauria	Cyanistes caeruleus	cyaCae2	28345	28345
Sauria	Gallus gallus	GRCg6a	28444	28444
Sauria	Gopherus agassizii	ASM289641v1	34329	34329
Sauria	Manacus vitellinus	ASM171598v2	26964	26964
Sauria	Melopsittacus undulatus	Melopsittacus_undulatus_6.3	22572	22572
Sauria	Nothoprocta perdicaria	notPer1	21931	21931
Sauria	Pelodiscus sinensis	PelSin_1.0	20669	20669
Sauria	Sphenodon punctatus	ASM311381v1	25260	25260
Sauria	Taeniopygia guttata	taeGut3.2.4	18204	18204
Sauria	Zonotrichia albicollis	Zonotrichia_albicollis-1.0.1	23611	23611
Mammalia	Bos taurus	ARS-UCD1.2	37538	37538
Mammalia	Callithrix_jacchus	ASM275486v1	40207	40207
Mammalia	Canis_familiaris	CanFam3.1	25158	25158

Mammalia	Carlito_syrichta	Tarsius_syrichta-2.0.1	31791	31791
Mammalia	Heterocephalus_glaber_male	HetGla_1.0	28099	28099
Mammalia	Homo_sapiens	GRCh38	110048	110048
Mammalia	Loxodonta_africana	loxAfr3	25635	25635
Mammalia	Macaca_mulatta	Mmul_8.0.1	45293	45293
Mammalia	Mesocricetus_auratus	MesAur1.0	25910	25910
Mammalia	Monodelphis_domestica	monDom5	22310	22310
Mammalia	Mus_musculus	GRCm38	67960	67960
Mammalia	Myotis_lucifugus	Myoluc2.0	20719	20719
Mammalia	Ornithorhynchus_anatinus	OANA5	23584	23584
Mammalia	Otolemur_garnettii	OtoGar3	19986	19986
Mammalia	Rattus_norvegicus	Rnor_6.0	29107	29107
